# Plasma Plasminogen Activator Inhibitor-1 Is Associated with End-Stage Proliferative Diabetic Retinopathy in the Northern Chinese Han Population

**DOI:** 10.1155/2012/350852

**Published:** 2012-10-14

**Authors:** Ze-Long Zhong, Song Chen

**Affiliations:** ^1^Clinical College of Ophthalmology, Tianjin Medical University, Tianjin 300020, China; ^2^Center of Vitreoretinopathy, Tianjin Eye Hospital, Tianjin 300020, China

## Abstract

*Objective*. To identify predictors of end-stage proliferative diabetic retinopathy (PDR) in a cohort of individuals with type 2 diabetes mellitus (T2DM) from the Northern Chinese Han population. *Methods*. We investigated characteristics of 153 consecutive diabetic patients with end-stage PDR (62 males, 91 females), 123 consecutive PDR patients without end-stage PDR (48 males, 75 females), and 151 normal subjects (63 males, 88 females). Only one eye of each patient or healthy subject was included in this study. Univariate logistic regression models and multivariate logistic regression models were constructed to evaluate the predictors of end-stage PDR. *Results*. In univariate analysis, systolic blood pressure, diastolic blood pressure, duration of diabetes, family history of T2DM, and plasminogen activator inhibitor-1 (PAI-1) were significently associated with end-stage PDR. After multivariate analysis, family history of T2DM, plasma PAI-1 levels, smoking, and duration of diabetes were four positive predictors associated with end-stage PDR. *Conclusions*. Higher plasma levels of PAI-1 were associated with end-stage PDR in the Northern Chinese Han population with T2DM.

## 1. Introduction

There has been much discussion recently about the risk factors for proliferative diabetic retinopathy (PDR). It remains uncertain what independent risk factors and predictors associated with end-stage PDR in the Northern Chinese Han population with type 2 diabetes mellitus (T2DM) exist. End-stage PDR is the most serious phrase of PDR. Diet, lifestyle, and depression [1] have been associated with a rapid increase in the incidence of DM in many countries throughout the world, particularly in developing countries such as China [2]. T2DM and its complications have been studied by many researchers in the fields of endocrinology [3, 4] and ophthalmology [5] since it is thought that identification and reduction of risk factors of DR may lead to a decrease of vision loss that is associated with this condition. Many previous studies have demonstrated that hereditary [6, 7] and environmental factors [8, 9] were both likely to be risk factors for PDR. Furthermore, it has been inferred that some cytokines in the peripheral blood might be predictors of end-stage PDR.

Systemic risk factors, including age, duration of diabetes, glycemic control, hypertension, renal impairment, the use of insulin, smoking, abdominal obesity, and genetic factors, have been reported to be associated with the progression of DR [10–15]. Additionally hyperopic refractive error [16] and axial length [17] are reported to be related to the development and progression of DR. However, whether there are circulating predictors of end-stage PDR in peripheral blood is unknown. The gene encoding human plasminogen activator inhibitor (PAI-1) was cloned from the lambda EMBL3 genomic library and was found to span approximately 12 kb and contain eight introns [18]. SERPINE1 (PAI-1) and other inflammatory mediators, such as ICAM1, IL-1, coagulation factor III, and VEGF, can cause retinal ischemia-reperfusion injury (RIRI) and retinal neovascularization [19]. The interaction between PAI-1 and TGF-*β* may ultimately induce the neovascularization seen in retinopathy of T2DM via the action of VEGF [20]. Therefore, it is plausible therefore that this circulating protein may predict the onset of end-stage PDR. Thus, targeting PAI-1 in addition to or instead of the traditional aforementioned risk factors may prevent progression to end-stage PDR. The aim of this study was to identify risk factors and predictors of end-stage PDR in a Northern Chinese Han population.

## 2. Methods

### 2.1. Study Design

From Jan 2009 to Jan. 2012, 153 consecutive patients with T2DM (62 males, 91 females) that had a clinical diagnosis of end-stage PDR were enrolled. 123 consecutive diabetic patients without end-stage PDR (48 males, 75 females) and 151 normal subjects (63 males, 88 females) also participated in the study. One eye, with the higher degree of damage in each patient, was included in this study when the severity of diabetic retinopathy in each of two eyes of T2DM was different. If two eyes were eligible, one eye was randomly included in this study using a randomization envelope. Control subjects were defined as the patients with PDR but without end-stage proliferative retinopathy. The eligibility criteria in the patients included known or newly diagnosed T2DM and age of 40 yr or older. Patients were excluded if they had acute complications of DM, type 1 or other types of DM, gestational diabetes, serious cardiovascular disease, hepatic, nephritic, or other complications, or other serious primary diseases or mental illness [9]. Individuals screened for the normal control group were excluded if they had any diseases of any system, as identified from their history and physical examinations [9]. 

### 2.2. Procedures

All examinations followed a similar protocol that was approved by the institutional human subjects committee of Tianjin Medical University and Tianjin Eye Hospital. The study was conducted in accordance with the Declaration of Helsinki. Informed written consent was obtained from all subjects enrolled in this study. 

The ocular and laboratory examinations included blood pressure, eye examination after dilating the pupils, stereoscopic color fundus photographs, family history of diabetes (yes or no), age, gender, duration of diabetes, duration of hypertension, systolic blood pressure (SBP), diastolic blood pressure (DBP), fasting blood glucose (FBG), postprandial blood glucose (PBG), blood urea nitrogen (BUN), creatinine (CREA), triglycerides (TGs), total cholesterol (TC), urine albumin, white blood cells (WBCs), red blood cells (RBCs), mean platelet volume (MPV), PAI-1 levels, and history of smoking. 

### 2.3. Definitions

T2DM was diagnosed according to the World Health Organization Expert Consultation Report [21] and consisted of one of the following: fasting blood glucose (FBG) ≥7.0 mmol/L, blood glucose ≥11.1 mmol/L 2 hours after an oral glucose tolerance test (OGTT), or random blood glucose ≥11.1 mmol/L. DR was clinically graded in accordance with the International Clinical Diabetic Retinopathy guidelines [22]. Early treatment diabetic retinopathy study (ETDRS) levels were as follows: 60–71, proliferative retinopathy (new vessels, vitreous haemorrhages, fibrovascular proliferation, and tractional detachment of retina) or scars of photocoagulation; 85, end-stage proliferative retinopathy (macula obscured by haemorrhage, retinal detachment at centre of macula, phthisis bulbi, or enucleation secondary to complications of diabetic retinopathy) [23]. The duration of diabetes was the period between the age at diagnosis and the age at the baseline examination [23]. Blood pressure was recorded twice with a random zero sphygmomanometer (Hawksley, Lancing, UK). Hypertension was defined as a systolic blood pressure ≥140 mmHg or diastolic blood pressure ≥90 mmHg and/or the use of blood pressure-lowering drugs [24]. A person was classified as never smoked if he/she had smoked fewer than 100 cigarettes in his/her lifetime and as a current smoker if he/she had not stopped smoking. Passive smoking was defined as in a previous study [25]. Controls were matched to end-stage PDR subjects based on age (≤5 years), gender, place of residency, and economical condition.

### 2.4. Laboratory Measurements

The plasma levels of PAI-1 were measured using ELISA arrays (Phoenix Pharmaceuticals, Inc., Belmont, CA, USA) according to the manufacturer's instructions. The measurement of plasma levels of PAI-1, BUN, CREA, plasma glucose, and triglycerides was done in accordance with the studies in [26–30].

### 2.5. Statistical Analysis

Continuous variables are presented as the mean with the standard deviation and were compared between end-stage PDR and the controls with a Student's *t*-test. Dichotomous variables were presented as percentages and were compared utilizing an *χ* ^2^ test. After one-way analysis of variance (ANOVA), the LSD test was used for the comparison among the mean values of three groups for different ages or plasma levels of PAI-1.

In the first part of the analysis, all potential risk factors including age, gender, type of diabetes, duration of diabetes, family history of diabetes, age, sex, duration of diabetes, SBP, DBP, FBG, PBG, BUN, CREA, TG, TC, urine albumin, WBC, RBC, MPV, PAI-1 level, and history of smoking (passive smoking was definited; active smokers with passive smoking were defined) were analyzed by univariate logistic regression analysis. The univariate relationships among the factors associated with PDR and the severity of PDR were analyzed with linear regression models. 

In the second part of the analysis, PAI-1 levels and other variables with *P* < 0.1 in the univariate analysis were included as independent variables in multivariate logistic regression models. Because the plasma PAI-1 levels were not normally distributed, they had been log transformed before the courses of multivariate logistic regression. We used the SPSS version 16.0 (SPSS Inc., Chicago, IL, USA) as the software for the statistical analyses. *P* < 0.05 was considered to indicate statistical significance. All *P* values were two sided.

## 3. Results and Discussion

In the present study, comparisons of baseline data between the patients with PDR and the normal subjects are shown in Table 1. The results from univariate logistic regression models demonstrated that SBP, DBP, duration of diabetes, family history of DM, PAI-1, FBG, MPV, CREA, and urine albumin are associated with the end-stage DR (Table 2). The results of multivariate logistic regression models demonstrated that patients who had a family history of T2DM, history of active smoking and passive smoking, and longer duration of diabetes were more likely to have end-stage DR (Table 3).

A striking finding from this study was that plasma levels of PAI-1 were an independent predictor of end-stage PDR in Northern Chinese Han patients with T2DM (Table 3). In our results, plasma levels of PAI-1 in patients with end-stage PDR were significantly higher than in patients without end-stage PDR or normals (Table 1). This is consistent with the fact that PAI-1 which is a risk factor for DR may serve as a useful marker of increased DR susceptibility [31]. Yan et al. reported that PAI-1 and other inflammatory mediators can lead to retinal ischemia-reperfusion injury (RIRI) [19] which is associated with oxidative stress. We therefore inferred that there might be a link between the activity of PAI-1 and oxidative stress in ocular tissues under hyperglycemic conditions. Consequently, elevated PAI-1 levels, a marker for underlying endothelial dysfunction, may be linked to an increased risk for diabetes [32] and might be a marker of diabetic microvascularization [31]. On the contrary, higher plasma levels of PAI-1 are reported to be independently associated with a lower risk of retinopathy [33]. Besides, Tarnow et al. reported that PAI-1 does not contribute to genetic susceptibility to DR [34]. The conflicting results between our studies and theirs might be related to differences in nationalities and the methodologies. Whether elevated PAI-1 levels are a significant biomarker of end-stage PDR must be testified by larger studies with subjects from different populations in the future.

The results of this study also suggested that family history of diabetes, smoking, and the duration of DM were independent risk factors of end-stage PDR in Northern Chinese Han patients with T2DM (Table 3). These are consistent with the fact that the previous studies reported [9, 35–40]. Therefore, genetic factors which were not measured easily might be an important risk factor of end-stage PDR. We also infer that smoking cessation and a reduction in exposure to smoke in people with T2DM may reduce the development of PDR and the onset of end-stage PDR. Besides, we consider that the length of duration of diabetes might predict the severity of PDR.

The first limitation of this study is that all patients were consecutively but not randomly selected. Our findings need further confirmation from larger numbers of randomly sampled cases. Second, the medications and treatment of the patients were not recorded in this study because medication and treatment conditions were not clear for some of the patients. In addition, the difference in nationalities and/or methods might have biased the results of this study to some extent relative to other studies.

In conclusion, our data suggested that PAI-1 levels are an independent risk factor of end-stage PDR. Because PAI-1 is associated with RIRI, which is associated with oxidative stress, we infer that targeting PAI-1 might prevent excessive oxidative stress in ocular tissues. Furthermore, assessing T2DM based on these identified independent risk factors, including PAI-1 levels, family history of T2DM, smoking, and duration of diabetes, might be a promising strategy to adopt to prevent the progression of PDR. Our results might provide novel approaches to the issue of preserving vision and preventing blindness.

## Figures and Tables

**Table 1:** Baseline data of patient characteristics in three groups.

	End-stage PDR		Normals (*n* = 151)
	Yes (*n* = 153)	No (*n* = 123)	
Male (*n*[%])	62 (40.523)^*⋇*^	48 (39.024)^*⋇*^	63 (41.722)
Age, years^#^	52.719 ± 6.661^*⋇*§^	57.626 ± 8.006*	53.136 ± 7.342
PAI-1, pmol/dL^#^	7.394 ± 2.084^∗§^	6.216 ± 0.989*	1.952 ± 0.276
95%CI	7.062–7.727	6.040–6.393	1.851–2.053
Family history of DM (*n*[%])	13 (8.497)^§^	36 (29.268)	—
History of hypertension (*n*[%])	89 (58.170)^§^	75 (60.976)	—
Smoking (*n*[%])			
Active	61 (39.869)	46 (37.398)	—
Passive	153 (100.000)	123 (100.000)	—
Duration of DM^#^	12.496 ± 6.173^§^	9.954 ± 4.441	—
FBG, mmol/L^#^	7.125 ± 1.987^§^	6.512 ± 2.157	—
PBG, mmol/L^#^	9.919 ± 3.401	9.226 ± 3.786	—
SBP, mmHg^#^	136.272 ± 11.114^§^	131.952 ± 14.719	—
DBP, mmHg^#^	84.771 ± 6.985^§^	82.602 ± 9.373	—
TC, mmol/L^#^	5.161 ± 0.784	5.222 ± 1.101	—
TG, mmol/L^#^	2.115 ± 1.509	1.854 ± 1.628	—
CREA, umol/L^#^	82.971 ± 52.230^§^	70.085 ± 33.491	—
BUN, mmol/L^#^	6.890 ± 2.630	6.345 ± 2.242	—
PAI-1, pmol/dL^#^	7.394 ± 2.084^§^	6.216 ± 0.989	—
WBC, 10^9^/L^#^	6.709 ± 1.606	6.513 ± 1.953	—
RBC, 10^12^/L^#^	4.350 ± 0.396	4.347 ± 0.563	—
HGB, g/L^#^	54.831 ± 65.599	130.452 ± 18.130	—
Lymphocyte counts, 10^9^/L^#^	7.643 ± 2.164^§^	1.784 ± 0.555	—
MPV, ^#^	9.871 ± 0.792^§^	10.192 ± 0.971	—

^∗^ *P* < 0.01 versus normals; ^§^ *P* < 0.05 versus no end-stage PDR; ^*⋇*^ *P* > 0.05 versus normal.

^#^Continuous variables are presented as the mean with the standard deviation.

**Table 2:** Univariate analysis of the progress of PDR.

	OR	*P* value	95% CI	
SBP	1.027	0.007	1.007	1.047
DBP	1.034	0.031	1.003	1.065
Duration of diabetes	1.914	*P* < 0.001	1.872	1.957
Family history of DM	7.446	*P* < 0.001	3.701	14.982
PAI-1	1.607	*P* < 0.001	1.329	1.942
FBG	0.869	0.017	0.775	0.975
PBG	0.948	0.115	0.888	1.013
TC	0 .932	0.588	0.723	1.202
TG	1.118	0.172	0.953	1.312
CREA	0.993	0.019	0.987	0.999
BUN	1.079	0.129	0.978	1.191
Urine albumin	0.734	0.004	0.594	0.907
WBC	1.068	0.406	0.914	1.249
REC	1.014	0.961	0.569	1.808
MPV	0.650	0.008	0.473	0.894
History of hypertension	0.933	0.802	0.545	1.600
Smoking	1.298	0.073	1.291	1.305

**Table 3:** Multivariate analysis of the progress of PDR.

	OR	*P*	95% CI	
Family history of DM	28.346	*P* < 0.001	5.554	144.678
Log value of PAI-1	3.113	0.001	3.066	3.169
Smoking	2.246	0.023	2.125	2.373
Duration of diabetes	1.748	*P* < 0.001	1.626	1.871
Urine albumin	0.141	0.103	0.013	1.484
CREA	0.937	0.001	0.902	0.973
FBG	0.573	0.001	0.690	1.320
MPV	1.393	0.520	0.409	0.803
SBP	1.024	0.383	0.971	1.080
DBP	1.135	0.051	1.029	1.251
